# Population Dynamics of the Exotic Flatworm *Obama nungara* in an Invaded Garden

**DOI:** 10.1002/ece3.70827

**Published:** 2025-01-16

**Authors:** Shanèze Noël, Yoan Fourcade, Virginie Roy, Georges Bonnet, Lise Dupont

**Affiliations:** ^1^ Univ Paris‐Est Créteil, Sorbonne Université, Université Paris‐Cité, CNRS, IRD, INRAE Institute of Ecology and Environmental Science, IEES Créteil France; ^2^ Amateur Naturalist La Rochelle France

**Keywords:** abundance, invasive species, *Obama nungara*, population dynamics, time series

## Abstract

Population dynamics and the way abundance fluctuates over time may be key determinants of the invasion success of an introduced species. Fine‐scale temporal monitoring of invasive species is rarely carried out due to the difficulties in collecting data regularly and over a long period. Thanks to the collaboration of an amateur naturalist, a unique dataset on the abundance of the invasive land flatworm *Obama nungara* was obtained during a 4‐year survey of a French private garden, where up to 1585 *O. nungara* were recorded in 1 month. Daily monitoring data revealed high population size fluctuations that may be explained by meteorological factors as well as intra‐ and inter‐specific interactions. Bayesian modeling confirmed that *O. nungara's* abundance fluctuates depending on temperature, humidity, and precipitation. Population growth seems to be favored by mild winters and precipitation while it is disadvantaged by drought. These exogenous factors affect both directly this species, which is sensitive to desiccation, and indirectly since they are known to affect the populations of its prey (earthworms and terrestrial gastropods). We also suggested the important resilience of *O. nungara* population in this site, which was able to recover from a drastic demographic bottleneck due to a severe drought, as well to systematic removal by the owner of the site. These findings highlight the potentially high invasiveness of *O. nungara* and raise concerns about the major threat these invasive flatworms pose to the populations of their prey.

## Graphical abstract

Fine‐scale temporal monitoring of the invasive land flatworm *Obama nungara* was obtained during a 4‐year survey of a French private garden, where up to 1585 *O. nungara* were recorded in 1 month. Population growth is favored by mild winters and precipitation while it is disadvantaged by drought. We higllighted the important resilience of *O. nungara* population in this site, which was able to recover from a drastic demographic bottleneck due to a severe drought, as well to systematic removal by the owner of the site, reaching a total cumulative abundance of 12,175 in 4 years.

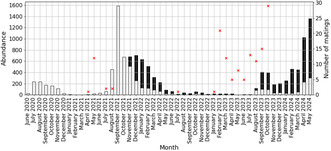

## 1 Introduction

Invasive species are among the leading causes of biodiversity loss (IPBES 2023) and may have detrimental impacts on invaded ecosystems at population, community and ecosystem levels (Vilà et al. 2011). Not all introduced species become invasive, though, and some established non‐native populations may persist at low abundance for a long period (years or even decades) and have negligible and/or undetectable impacts. During this lag phase, an alien species may appear to be a low threat to the invaded ecosystem, while it is actually acclimatizing to its new community (Crooks 2005; Haubrock et al. 2024). Environmental factors may trigger population growth, such that it becomes rapidly highly abundant and can start to cause significant impact (Spear et al. 2021). For instance, increased abundance of alien predators is linked to a strong decline of native population size and native community richness and diversity (Bradley et al. 2019). According to the stage‐based framework of the invasion process, an increase of abundance is the signal that an alien species has entered the proliferation and spread stages (Blackburn et al. 2011; Daly et al. 2023). After demographic explosion, the speed of invasion spread will be influenced by the population growth, abundance, and dispersal of the species, which depend on habitat characteristics (Goldstein et al. 2019). Thus, internal population dynamics and the way an invasive species' abundance fluctuates are key determinants of the invasion trajectory (Bradley et al. 2019; Haubrock et al. 2022).

Population trajectories may be investigated using abundance‐based time series that must include as many years as possible because short‐term observations may lead to potentially misleading conclusions about the species' population dynamics (Haubrock et al. 2024). However, temporal changes in abundance of invasive species are rarely investigated, as most invasive species monitoring and databases emphasize on occurrence rather than abundance (Hansen et al. 2013). This is largely due to the difficulty of obtaining precise temporal abundance data, over a sufficiently long period, at a given site. Data from participatory monitoring offer a great potential to obtain this kind of information and to detect temporal biodiversity trends (Mandeville et al. 2023; Pocock et al. 2024). Although citizen science data generated by large monitoring networks generally present various biases (Kosmala et al. 2016), smaller scale observations supported by a single or a small group of motivated volunteers may be particularly beneficial to acquire data (i) at various times of the day, (ii) over a long period of time and (iii) during an extended period of the year.

**FIGURE 1:**
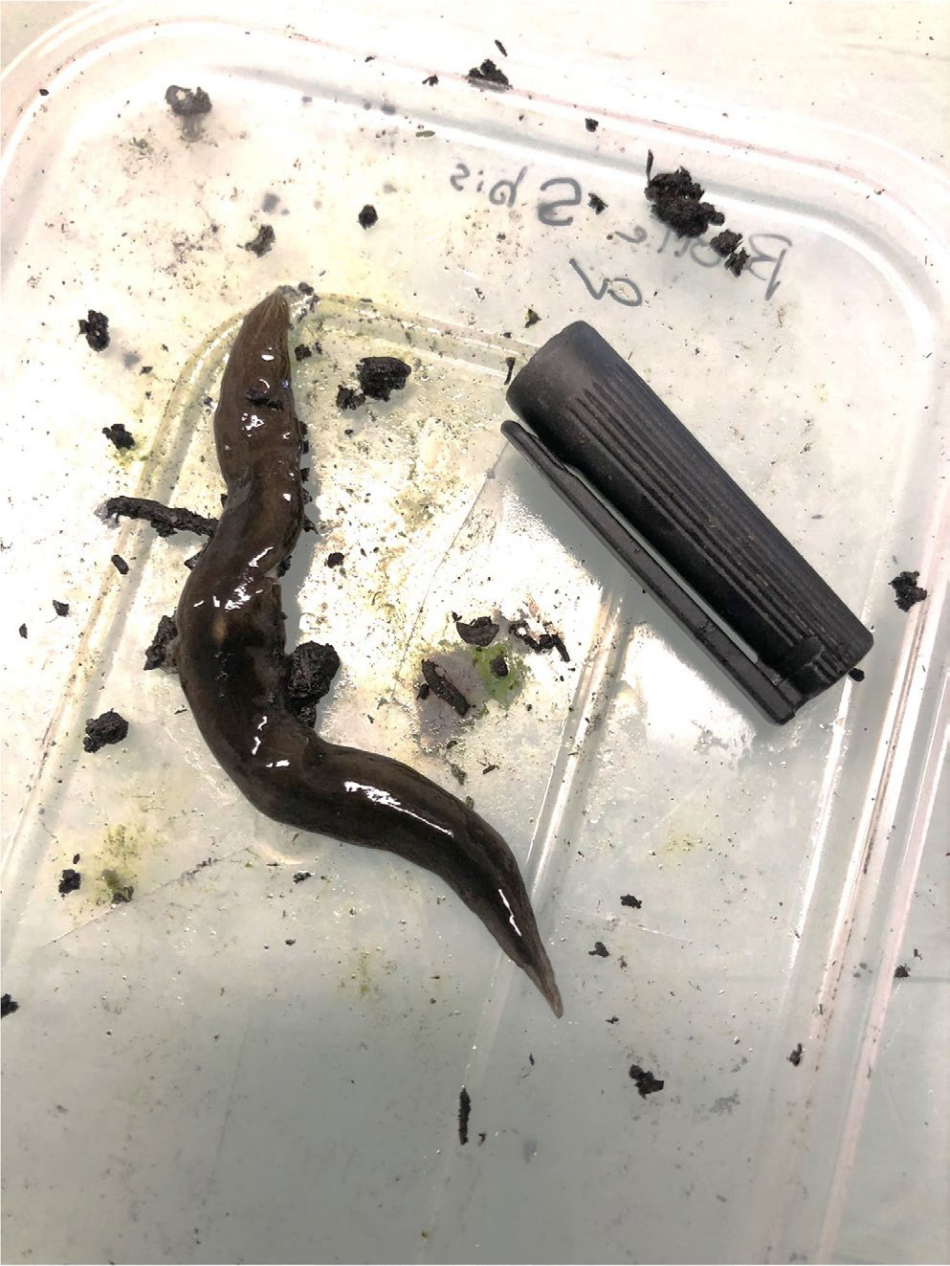
Photography of an *Obama nungara* in the laboratory next to a 4‐cm marker cap for scale.

Citizen science programs provided valuable information to monitor the spread of invasive land planarians (Platyhelminthes, Geoplanidae) in metropolitan France and French overseas territories for more than 10 years (Justine et al. 2018, 2020). Among them, *Obama nungara* (Figure 1) is the most invasive land flatworm in Europe in terms of its area of invasion and population density in invaded areas (Justine et al. 2020). This species native to South America has successfully spread across numerous countries since its first European report in 2008 in Guernsey Island (Carbayo et al. 2016; Justine et al. 2020; Lazányi et al. 2024; Negrete et al. 2020). Because land planarians and their cocoons can easily travel in pot plants, *O. nungara* was probably transported from its native range, and spread among introduced areas, through the trade of plants (Justine et al. 2020; Sluys 2016). The broad diet niche of this top predator that feeds mainly on earthworms but also on potworms, snails, slugs, and other land planarians (Boll and Leal‐Zanchet 2016; Roy et al. 2022) contributes to the success of its establishment in introduced areas and suggests that this species has a great potential to threaten the stability of the invaded ecosystems. Modeling revealed that the potential distribution of *O. nungara* was mainly outlined by climatic factors related to temperature (Fourcade 2021; Negrete et al. 2020) while it is also known that *O. nungara* is strongly dependent on a moist environment (Boll and Leal‐Zanchet 2018).

Although this species has occasionally been reported in natural environments (Lago‐Barcia, González‐López, and Fernández‐Álvarez 2020), it is principally found in anthropized areas such as gardens and parks (Justine et al. 2020; Lago‐Barcia et al. 2019), a characteristic facilitating its monitoring by citizen science. Thus, since its first report in 2013, the spread of *O. nungara* in France was monitored thanks to a citizen science network during 5 years (Justine et al. 2020). This survey revealed that, in 2018, *O. nungara* was already particularly widespread, having invaded 75% of the metropolitan territory, and it locally reached high abundance, with hundreds of specimens recorded in a single garden (Justine et al. 2020). During this survey, reports of *O. nungara* were not regular, with more reports in spring compared to autumn and minimal reports in winter. This variation in the number of reports over the course of a year could be explained by either seasonal variability in abundance or by periods when citizens were more active (i.e., periods when gardeners were spending time in their garden or when the media were communicating about this research), which are temporal biases inherent in citizen science data (Justine et al. 2020).

Here, we analyzed a unique dataset on the abundance of *O. nungara*, where individuals were collected and removed daily by a citizen volunteer at a single site over a period of 4 years. This dataset provides an opportunity to better understand the invasiveness of *O. nungara* in terms of one of the drivers of invasion success, population growth in the introduced range. Specifically, our main objective was to examine the temporal variability of *O. nungara* population abundance at a single site in order to (i) explore the seasonal variation in abundance during a year, (ii) examine the spatial variation in abundance within a garden and (iii) determine how climatic variables (i.e., temperature, humidity and precipitation) affect *O. nungara* population dynamics.

## 2 Materials and Methods

### 2.1 Study Site and *O. nungara* Daily Counting

The study was carried out in a garden of 1275 m^2^ located in La Rochelle, France, where the climate is coastal and oceanic. The owner has volunteered to prospect for *O. nungara* in his garden after its participation in the citizen inventory of alien flatworms initiated by the MNHN (Museum National d'Histoire Naturelle). The site is a recreational garden planted with ornamental plants, which is frequently visited by two people, including the owner, and is also used for gardening. No other use has been reported, and three domestic cats live in the garden as well as some wild hedgehogs. Data were collected daily from June 10, 2020 to May 31, 2024. Overall, data were collected on 1260 days out of 1452 days in this 4‐year period with an interruption of 6 weeks in February and March 2021 during which the owner was unable to count the flatworm in his garden. The owner aims to maximize the number of *O. nungara* sampled each day in his garden. At the start of the survey, he only sampled during the day, then from November 2021 he started to sample at night too. The total time spent sight‐hunting was adjusted according to the number of *O. nungara* found. Spending more time looking for them when numbers were large. In total, the duration can therefore range from 1 h to 3 h with one sampling session during the day (at noon) and one to two sessions at night (from 10 p.m. to midnight and in winter also at dusk around 5 p.m.). He was looking for *O. nungara* in any habitat that could host it (rocks, plant pots and wood lying on the ground). All *O. nungara* observed were counted, their geographical location was noted and they were preserved in ethanol. Mating events were also recorded opportunistically. Each time a new habitat was discovered, the location was checked daily onwards. Twice a year in autumn and in spring, our team carried out fieldwork on the same site to confirm the presence of the species, collect additional data and discuss the owner's data collection protocol.

### 2.2 Spatial Analysis of *O. nungara* Spread

We used Google Earth Pro Software (Version 7.3.6.9345) to pinpoint GPS locations of each habitat. R software (Version 4.3.0) (R Core Team 2023) was used to display the spread of *O. nungara* across the garden. To do so, we used the “OpenStreetMap” (Fellows and Stotz 2023) and “ggh4x” (van den Brand 2024) packages to map the locations occupied by *O. nungara* and its total abundance throughout time, drawing two seasonal maps per year (spring and autumn). We chose this time step because it fitted our estimation of flatworms' reproduction periods and showed the most variation without impairing visibility on the map.

### 2.3 Temporal Analysis of *O. nungara* Abundance

To determine how weather may contribute to abundance fluctuation and population dynamics of *O. nungara* over time, we downloaded temperature, humidity and precipitation data from the nearest open access "Météo France" station, which is the "7314‐Porte de Chassiron" station (Latitude: 46.0466917, Longitude: −1.41) located on Oleron Island close to the coast of La Rochelle, approximately 25 km away from our study site. We checked that weather variables were only little correlated, using both correlation graphs, made using "corrplot" package (Wei et al. 2021) and Pearson's correlation tests (maximum correlation: temperature and humidity, *r* = −0.223, *p* < 2.2 × 10^16^). We plotted daily *O. nungara's* abundance and one weather variable per graph using Python software (Version 3.10.4) (Van Rossum and Drake 2009) and packages "pandas" (version 1.4.4) (McKinney and Others 2010) and "matplotlib" (version 3.7.1) (Caswell et al. 2023). For humidity and temperature variables, we used a kernel density estimation (KDE) smoothing function which allows us to visualize variation over time more easily than plotting daily data. However, this was not appropriate for precipitation, since smoothing would obliterate heavy rainfall events.

Population dynamics was analyzed using zero‐inflated Poisson regressions, as it is most appropriate for count data (Winter and Bürkner 2021). Models were fitted using Bayesian inference, which allows to estimate parameters as posterior distributions instead of point estimates, and which are especially used for the study of single‐species population dynamics (Ellison 2004). We used "brms" and "stan" packages (Bürkner et al. 2023), with four chains of 2000 iterations and weakly informative priors. First, we established a model focusing on the influence of same‐day weather on abundance. Because the effect of lack of precipitation can be seen after a few days, weeks or even months depending on the amount of residual water present in the soil, or soil porosity, and the evaporation and redeposition of water controlled by temperature and air humidity, we also tested if there might be some lagged effects of weather variables on abundance (i.e., weather of the previous weeks may have an impact on abundance). Thus, we calculated the moving average of each variable on different time scales: 3 days, a week (7 days), a month (30 days), 3 months (91 days, season equivalent) and a year (365 days). The package "imputeTS" (Moritz et al. 2022) was used to fill in missing weather data (percentage of missing data: 1.7% for temperature and humidity, 2.1% for precipitation). Here, the missing weather data were filled with the average of the previous and following day. Then, we implemented the model with *O. nungara* counts (until May 1, 2024) as response variable and the lagged weather data as explanatory variables. We also included as covariables the year, the period of collection (day or night), as well as the day of the year fitted as a cyclic penalized cubic regression spline smooth. Finally, we added the identity of the habitat as a random intercept. In total, we fitted six models with six different time frames. Posterior predictive plots, R hat criterion, and trace plots were used to check model fit, convergence, and chain mixing. We used approximate Leave‐One‐Out cross validation (LOO) to estimate which model fits best the data. On the selected model, we performed a temporally structured 12‐fold cross‐validation in which data were partitioned by month (January to December). Therefore, each month was used in turn to test model fit while the remaining 11 months were used for model training. We then calculated a Bayesian measure of *R* ^2^ for each test set (Gelman et al. 2019) and reported the mean and standard deviation of *R* ^2^ across the 12 folds. Mean estimates and 95% credibility intervals were reported for each variable and each imputed time scales (same day, 3 days prior, a week prior, a month prior, a season prior, and a year prior) (Figure 5, Table 2).

## 3 Results

### 3.1 Spatial Analysis of *O. nungara* Spread

**FIGURE 2:**
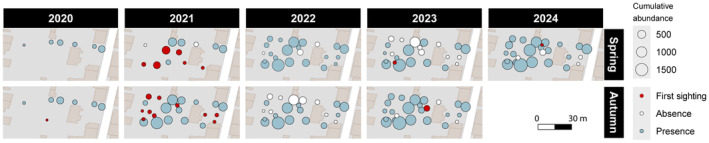
Expansion map of *Obama nungara* in the garden over time with a 6‐month step. Cumulative abundance per location is displayed via the size of each circle. New locations are shown in red, locations where no *O. nungara* was found in the 6‐month period are shown in white.

The expansion map of *O. nungara* revealed that, in spring 2020, *O. nungara* was already found in six locations and was found in a seventh location in Autumn 2020 (Figure 2). Locations are different types of microhabitats at least 1 m away from each other. From this period onwards, a large number of individuals will regularly be recorded at a specific location until reaching a total cumulative number of 1937 *O. nungara* found in this single location (Table 1). In autumn 2021, most of the garden was colonized, with 16 more sites newly occupied, during the 6 months, and the abundance rising over 500 individuals in the most central locations. In 2022, nine sites were no longer showing signs of *O. nungara* presence. In 2023 and 2024, we noticed that almost all the sites where nothing was found in 2022 are re‐colonized. Only four sites out of 25 completely stop presenting *O. nungara* in the 4‐year span (Figure 2). One new location appeared regularly every season in the last year and a half.

**TABLE 1:** *Obama nungara*'s cumulative abundance per location over the 4 years of sampling.

Location	Abundance
A	306
B	96
C	880
D	436
E	1098
F	581
G	1246
H	209
I	1417
J	1066
K	513
L	1937
M	265
N	96
O	78
P	13
Q	39
R	259
S	235
T	383
U	65
V	234
W	124
AA	268
AB	304
AC	27
Total	12,175

### 3.2 Temporal Analysis of *O. nungara* Abundance

**FIGURE 3:**
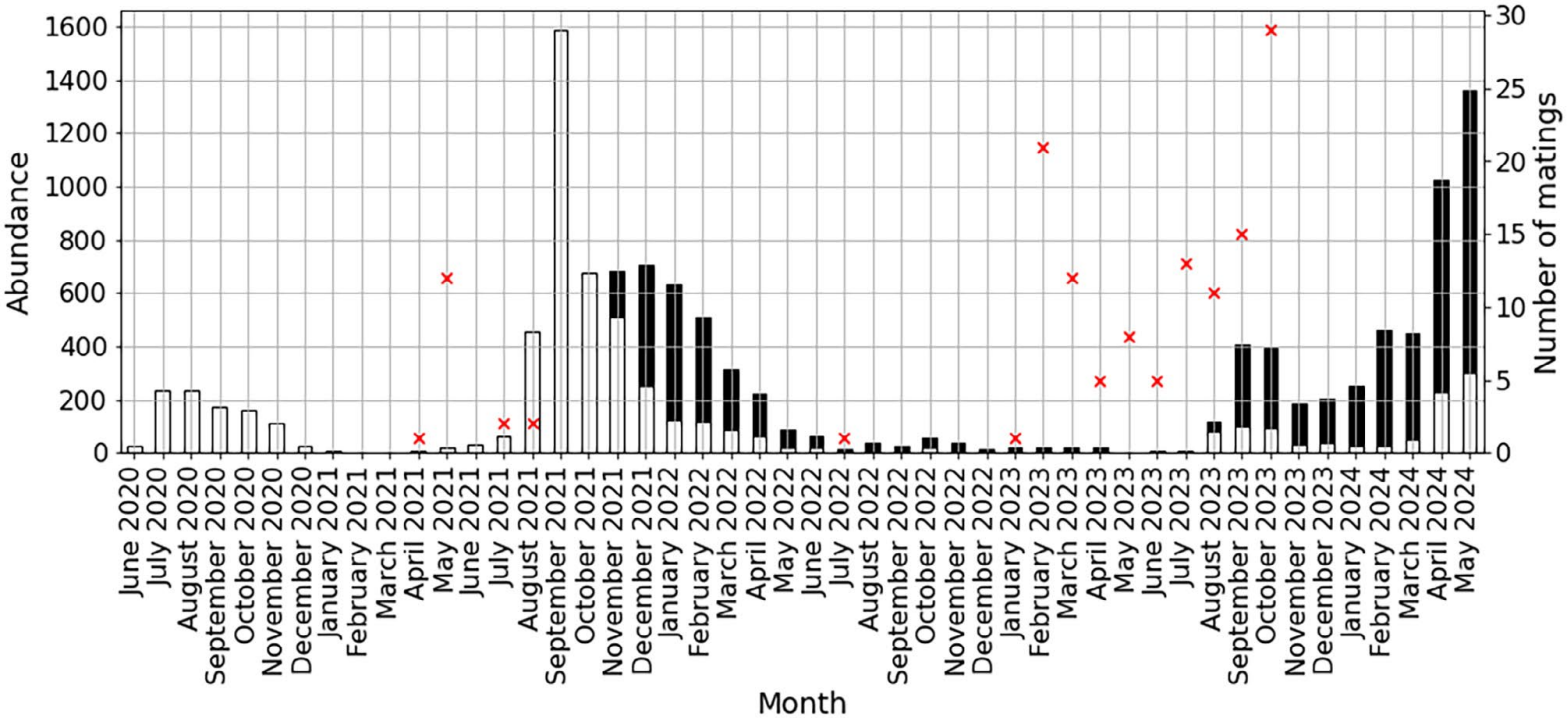
*Obama nungara*'s abundance per month over time. Day abundance is displayed in white and night abundance is displayed in black. The night counts began in November 2021. Number of mating events is displayed with red crosses.

A seasonal fluctuation of *O. nungara*'s abundance was observed with maximum abundance occurring in late summer or up until late winter (Figure 3). In November 2021, the owner began night counts, more *O. nungara* were found at night than during the day. The model revealed that the probability of finding a higher abundance of *O. nungara* at night is 0.83 (credibility interval: 0.78–0.88). Mating started being seen consistently in February 2023, then mating reports stayed over 10 months until October 2023 when over 25 mating events were reported (Figure 3).

We noticed three peaks of *O. nungara's* abundance during the survey period (Figure 3): a small peak in early summer 2020 which continued until mid‐autumn 2020 with a maximum of 235 *O. nungara* in August 2020. In 2021, numbers remained low until August 2021 when abundance started to increase again, with a record month registering 1585 *O. nungara* in September 2021. The peak declined very gradually until April 2022 (220 *O. nungara* found). Then, until July 2023, the monthly counts of *O. nungara* were all below 100. However, in August 2023, the number of *O. nungara* increased again and remained high until October 2023. In both September and October 2023, around 400 *O. nungara* were found. The abundance continued to rise until it reached its maximum, for this year in April 2024 with 1026 *O. nungara*. In total, 12,875 *O. nungara* were found at this site during the 4‐year period. The population density of *O. nungara* in this garden reached 1 ind./m^2^ in the months where the flatworms were most abundant.

**FIGURE 4:**
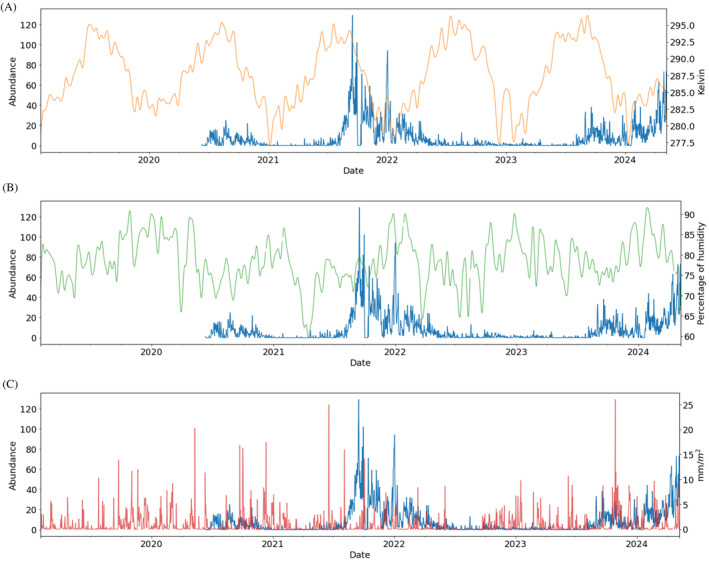
*Obama nungara*'s daily abundance over time (in blue) and weather variables. (A) Temperature (K) in yellow with KDE smoothing, (B) humidity (%) in green with KDE smoothing, and (C) precipitation (mm/m^2^) in red.

A seasonal periodic variation in temperature was observed (Figure 4A) with a higher peak in summer 2022 than in the other years, the maximum temperature being 28.2°C (301.3 K) and repeated twice in a fort night, whereas summer 2021 and summer 2023 peaked at 24.7°C (297.8 K) and 26.2°C (299.3 K), respectively. In the winter, there were no records of negative temperatures (lowest temperature: 0.5°C (273.6 K)); thus, the ground never freezes.

Humidity was also rather cyclic every year with a decrease in summer (Figure 4B), except in 2023 where humidity remained high throughout summer. Average daily humidity per year was usually around 78.4% (SD = 10.2). In 2019, we were at a maximum with 79.7% (SD = 9.7) average daily humidity and 2021 was the driest year with an average daily humidity of 75.8% (SD = 10.2).

Precipitation occurred regularly with rain at least once a month except in the summer (Figure 4C), in July especially where rain seemed to stop for over a month. However, we noticed that the third trimester of 2022 was especially dry with almost no precipitation for 3 months, in total there was only 6 mm/m^2^ of rain. Moreover, 2022 is the only year not displaying a peak in precipitation (above 20 mm/m^2^). Indeed, three peaks of precipitation can be seen throughout the years on May 11, 2020 (20 mm/m^2^ of rain in a day), June 18, 2021 (25 mm/m^2^ of rain in a day), and October 28, 2023 (26 mm/m^2^ of rain in a day).

**FIGURE 5:**
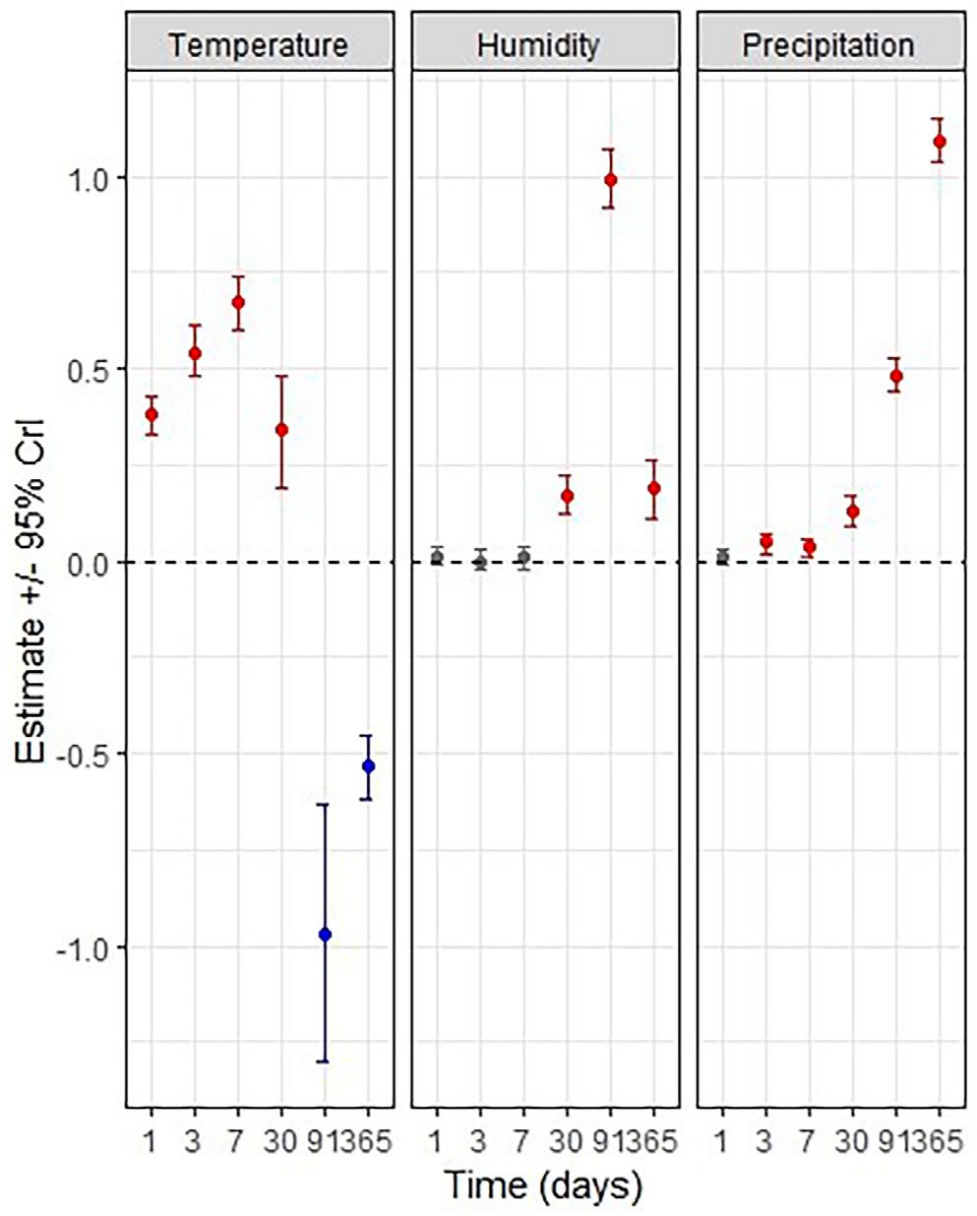
Estimates of the effect of each variable (temperature, humidity, and precipitation) on *Obama nungara* abundance, shown as the mean of posterior distributions with 95% credible intervals. Points in blue and red highlight, respectively, a negative and positive estimated effect of the variable on abundance (i.e., 95% CrI that do not cross 0); in gray are shown variables for which the direction of their effect cannot be ascertained (i.e., 95% CrI that contain 0).

In short time scales (i.e., same‐day, 3 days and week), we did not find an evidence for an effect of humidity (Figure 5 and Table 2). Whereas humidity seemed to have positive impact on abundance in long time scales (i.e., humidity of the previous month, season, and year seems to impact abundance positively) (Table 2). Temperature seems to be positively associated with *O. nungara* abundance in the short time scales whereas in the long times scales, it seems to be strongly negatively associated with *O. nungara* abundance (Figure 5 and Table 2). Precipitation has a strong positive association in all models except “same‐day” (Table 2). Comparing the six models using approximate LOO cross‐validation revealed that the best fitting model was achieved with the variables representing the mean value over the 365 preceding days. Cross‐validation tests revealed a mean *R* ^2^ of 0.13 (standard deviation = 0.11), for this model.

**TABLE 2:** Estimates of the effect of the three climatic variables on *Obama nungara*'s abundance, shown for each model along with confidence intervals and standard errors.

Model	Variable	Estimate	Lower confidence interval	Upper confidence interval	Standard error
1	Temperature	0.38	0.33	0.43	0.03
	Precipitation	0.01	−0.01	0.03	0.01
	Humidity	0.01	−0.01	0.04	0.01
3	Temperature	0.54	0.48	0.61	0.03
	Precipitation	0.05	0.02	0.07	0.01
	Humidity	0.00	−0.02	0.03	0.01
7	Temperature	0.67	0.60	0.74	0.04
	Precipitation	0.04	0.01	0.06	0.01
	Humidity	0.01	−0.02	0.04	0.02
30	Temperature	0.34	0.19	0.48	0.07
	Precipitation	0.13	0.09	0.17	0.02
	Humidity	0.17	0.12	0.22	0.02
91	Temperature	−0.97	−1.30	−0.63	0.17
	Precipitation	0.48	0.44	0.53	0.02
	Humidity	0.99	0.92	1.07	0.04
365	Temperature	−0.53	−0.62	−0.45	0.04
	Precipitation	1.09	1.04	1.15	0.03
	Humidity	0.19	0.11	0.26	0.04

## 4 Discussion

In this study, we were able to collect detailed data on the temporal and small‐scale spatial dynamics of a population of the invasive flatworm *O. nungara* in a single private garden. This unique dataset was obtained thanks to the commitment of the site owner who collected data daily, sometimes even thrice a day, over a 4‐year period, providing thus a valuable temporal dataset for monitoring changes in *O. nungara* abundance. This volunteer also had extensive knowledge of his garden management, giving the scientists access to unique information about the environmental conditions of their study. Finally, such data would have been particularly difficult to obtain using a scientist‐only approach due to the extensive funding required for this type of fieldwork. Here, we highlight that increased temporal coverage, cost‐effectiveness, enhanced sampling frequency and access to unique insights are key benefits of citizen science for invasive species research (Pocock et al. 2024).

We showed that *O. nungara* was able to successfully spread until it invades a large part of the garden in 4 years. Source–sink population dynamics might be hypothesized to explain such invasive spread (Thomson 2007). In the hypothesis that the “sources” that support local population growth are outside the garden, the population within the garden would be the “sink” where mortality would exceed natality and therefore could not sustain local population without immigration. However, our results show on the contrary that, after an initial introduction presumably from the garden's entrance (upper right corner) (Figure 2) in 2020 or before, the greatest abundances were reached in the central sites, 4 years later. The population of the garden therefore does not seem to be supplied by external sources, although we cannot exclude secondary introductions through human activities via gardening supplies (e.g., substrate, fertilizer, plants). To the contrary, because this population reached very high abundance, which demonstrate that over the entire period studied the birth rate was higher than the mortality rate, this garden could be a source for the colonization of adjacent gardens. However, to date, no flatworms have been reported in neighboring gardens. This can be explained by the presence of 2 m high stone walls demarcating the entire garden, which flatworms are unlikely able to cross over without desiccating first. This brings out serious concern about their spreading dynamics in a non‐limited area.

The data collected as part of this study made it possible not only to describe the spread of *O. nungara* in the garden over time but also the fluctuations in population abundance. The abundances observed per month range from 3 to a record of 1585 flatworm in September 2021 which correspond to an approximate density of 1.24 *O. nungara* per m^2^ (using the surface of the whole garden). During this 4‐year survey, three peaks of abundance were observed. Fluctuations in population size is a well‐known process in ecological theory (Bjørnstad and Grenfell 2001; Hastings 2004), that may be caused by both intra‐ and inter‐specific interactions, as well as purely exogenous factors such as weather (Pisa, Hermisson, and Polechová 2019). Here, we will discuss specifically four kind of non‐exclusive factors that may explain the observed pattern: (i) meteorological factors, (ii) predator–prey interactions, (iii) intraspecific factors (density dependence and founder effects), and (iv) human action on the population.

The first peak of abundance was observed from approximately July 2020 to November 2020. During the winter 2020–2021 and the spring 2021, abundance was very low. This is coherent with the observations reported in Justine et al. (2020) who proposed that *O. nungara* population decreases in winter. We may indeed expect that during cold periods, the activity of the flatworm is slowed down. Nevertheless, in this geographical area, winter is in general mild with no sub‐zero temperatures recorded during the study period, so the ground most likely never froze which is an advantage for the survival of the flatworm and its prey. In 2021/2022, *O. nungara* was abundant during a very large part of the year with a peak of abundance spanning approximately from August 2021 to April 2022. The fact that abundance did not strongly decrease in winter 2021–2022 could be explained by a winter particularly mild, compared to other winters of the period studied, with temperature ranging from 0.5°C to 14.2°C from December 2021 to March 2022 (Figure 4A). We indeed showed that temperatures have a positive impact on the flatworm's abundance in short times scales (Figure 5). In 2023–2024, a small decrease in abundance was observed in early Autumn but significant numbers of flatworms continued to be observed with an abundance of 251 *O. nungara* during the colder month January 2024 with a monthly average of 8.1°C (SD = 4.1). It therefore appears that, in this geographic region, winter does not have a particularly strong negative effect on the survival and activity of *O. nungara*.

During the period from July 2022 to July 2023, abundance was extremely low with no resurgence of an abundance peak in August 2022 as the previous peaks might suggest. At that time, the *O. nungara* population appeared to be on the verge of extinction. Indeed, in an earlier survey, some citizens mentioned the disappearance of this species from certain gardens where it was previously abundant (Justine, Gastineau, and Winsor 2024). However, from August 2023 onwards, abundances increased again. These observations can probably be explained by the severe drought that occurred during the summer 2022, the total precipitation from July to September 2022 was 6 mm/m^2^. The average summer over the last 5 years had 79.7 (SD = 40.4) mm/m^2^ of precipitation throughout summer. The main causes of drought are water deficit and high temperatures, and we demonstrated that lower precipitation was associated with reduced abundance which is stronger at larger time scales while temperature have a strong negative effect on flatworm's abundance in large time scales (Figure 5). Every big peak of *O. nungara*'s abundance was preceded (i.e., in the previous month) by a huge rainfall event (over 20 mm/m^2^ of rain, Figure 4C) showing the delayed impact of precipitation on abundance. We also notice that air humidity positively correlates with *O. nungara* abundance on time scales larger than a month. It seems coherent that long‐lasting high humidity, for at least a month, is necessary to have an impact on a soil invertebrate such as *O. nungara* whose survival depends on a wet environment. Indeed, this species is prone to desiccation on a dry medium (Boll and Leal‐Zanchet 2018) and the presence of its prey (earthworms, snails and slugs) is favored by humidity.

The main preys of *O. nungara* are soil invertebrates and the fluctuations of *O. nungara* abundance could also be explained by the influence of the climatic conditions on the abundance of its prey. For instance, it is known that earthworm abundance tends to decline in the dry or very cold season and reaches highest densities and biomass when climatic and soil conditions are more favorable, which are conditions typically occurring in spring and autumn in temperate regions when temperature is modest and soil water content is high (Singh et al. 2019). Various climatic factors like temperature, precipitation, soil moisture, as well as extreme climate events like drought have been shown to alter the composition and functioning of communities in the soil (Singh et al. 2019). For instance, the drought observed in summer 2022 in the study area has certainly reduced the biological activity and earthworm biodiversity in soils thus reducing the number of prey available to *O. nungara*. Some land snail species are known to have behavioral and physiological adaptations that allow individuals to survive in very dry and hot environments, but these could be associated with reduced accessibility to predators such as *O. nungara*, for example, burrowing and climbing (Schweizer, Triebskorn, and Köhler 2019).

In addition to meteorological factors, fluctuation of abundance of this invasive species may also be explained by intraspecific biological factors. It is well known in ecology that intrinsic density feedbacks can cause population densities to fluctuate, even in constant environment (May 1974). Such density dependence plays an important role in population regulation but the ways it affects the process of invasion is less understood (Sullivan et al. 2017). For *O. nungara*, likely density‐dependent factors include prey availability and intraspecific competition. Among the eco‐evolutionary processes that affect the population dynamics of an invasive species once established is also the founder effect which describes the reduction in genetic diversity in an invasive population, relative to the parent population, due to a small number of founding individuals (Sakai et al. 2001). The potential implications of founder effects for newly invasive populations include inbreeding depression, reduced adaptive capacity and/or high extinction risk, which may slow the range expansion of the invasive species (Hagan et al. 2024). In the study site, the population has experienced a drastic demographic bottleneck in 2023 which could have been important for population dynamics by exacerbating the effect of a potential already lowered genetic diversity in the population. Nevertheless, the population recovered well from this bottleneck and reached again high abundance in summer 2024 highlighting that *O. nungara* introduced population have the ability to grow despite population bottlenecks. The fact that bottlenecked populations that typically have low genetic diversity, low evolutionary potential and perhaps low reproductive fitness can become invasive is a genetic paradox that have received much attention in invasion biology (Frankham 2005).

A factor that is likely responsible of a regular decrease of *O. nungara* population size in the study site is the human factor. When animals are searched for and killed systematically in an enclosed space, we can assume that their numbers will drastically reduce until extinction. Here, every *O. nungara* seen and counted by the owner was removed from the garden and killed. During the study, the owner even intensified his search by searching for the *O. nungara* both day and night. Despite this, high *O. nungara* abundance was still observed in spring 2024. Two non‐exclusive hypotheses arise to explain the population's resilience to systematic killings. First, by decreasing population size, human action would decrease the intraspecific competition which would have, in a context of density‐dependent regulation, a positive effect on *O. nungara* abundance. Second, *O. nungara* flatworms may benefit in the study site from permanent shelters, allowing them to hide from humans and adverse climate. Although a significant number of flatworms are killed every day, we can hypothesize that a very large number live hidden and unreachable. For instance, they could be hidden under the huge stone terrace of the garden, located near the center and where most *O. nungara* are found, where some *O. nungara* were seen crawling out from under the terrace. This terrace may provide a permanent shelter where *O. nungara* could hunt and reproduce away from any disturbance.

## 5 Conclusion

In this study, we showed that *O. nungara* can spread rapidly in a garden, despite temperature, humidity, and precipitation affecting its presence. Although the population seemed to have dramatically declined after an initial outburst, it was able to recover from this demographic bottleneck. Thus, the flatworms are still present despite extensive effort by the owner to kill them. In May 2024, the abundance reached 1360 individuals. This suggest the potentially high invasiveness of *O. nungara*. The impact on the communities of their prey may be huge. As one adult *O. nungara* eats on average six preys a month (Unpublished laboratory observation), this roughly correspond to 8160 preys during the month of May 2024. High abundance combined with their food intake could have dramatic effect for native soil fauna in their introduced range. Further studies will investigate the impact of *O. nungara* on its preys in metropolitan France, in particular on earthworm communities, and the consequence of this predation on soil ecosystem functioning.

## Author Contributions

**Shanèze Noël:** conceptualization (lead), data curation (supporting), formal analysis (equal), methodology (lead), writing – original draft (lead). **Yoan Fourcade:** formal analysis (equal), methodology (equal), validation (lead), writing – review and editing (supporting). **Virginie Roy:** conceptualization (supporting), methodology (supporting), supervision (supporting), validation (supporting), writing – review and editing (supporting). **Georges Bonnet:** data curation (lead). **Lise Dupont:** conceptualization (equal), funding acquisition (lead), project administration (lead), supervision (lead), validation (lead), writing – review and editing (lead).

## Conflicts of Interest

The authors declare no conflicts of interest.

## Data Availability

The data that support the findings of this study are available on Figshare DOI: 10.6084/m9.figshare.27043804. No GPS locations or coordinates are published due to reasons of privacy. They are available from the corresponding author upon reasonable request. These data are located in controlled access data storage at University Paris Est Créteil.
